# Frailty and its association with long-term mortality among community-dwelling older adults aged 75 years and over

**DOI:** 10.1186/s13584-024-00614-y

**Published:** 2024-07-16

**Authors:** Maor Lewis, Anthony Heymann, Galia Zacay, Dan Justo

**Affiliations:** 1 Department of Family Medicine, Meuhedet Health Maintenance Organization, Tel-Aviv, Israel; 2 Faculty of Medicine and Health Sciences, Tel-Aviv University, Tel-Aviv, Israel; 3 Division of Geriatrics, Sheba Medical Center, Tel-Hashomer, Ramat Gan, Israel

**Keywords:** Community-dwelling, Aging, Cumulative deficit method, Frailty, Mortality, Older adults

## Abstract

**Background:**

Frailty, a significant risk factor for adverse outcomes and mortality, poses an emerging challenge with profound implications for public health and clinical practice. The measurement of frailty offers potential enhancements in healthcare services for older adults. The prevalence of frailty and its association with long-term mortality in a nationwide, unselected population of community-dwelling older adults, particularly those aged 75 and over, has not been previously studied on a large scale in Israel.

**Methods:**

A retrospective cohort study was conducted at Meuhedet Health Maintenance Organization, Israel’s third largest healthcare service provider, serving 1,276,000 people (13.8% of Israelis). The prevalence of frailty and its association with all-cause mortality were studied among older adults aged 75 years and over who were followed for 2–8 years. Frailty, defined by the cumulative deficit method, utilized clinical data from the preceding 10-year period, comprising 28 chronic diseases and age-related health deficits.

**Results:**

The cohort included 43,737 older adults, with a median age of 77 years (IQR 75–82 years); among them, 19,300 (44.1%) were males. Overall, 19,396 (44.3%) older adults were frail: 12,260 (28.0%) mildly frail, 5,533 (12.7%) moderately frail and 1,603 (3.7%) severely frail. During the follow-up period 15,064 (34.4%) older adults died: 4,782 (39.0%) mildly frail, 3,016 (54.5%) moderately frail and 1,080 (67.4%) severely frail. Cox regression analysis demonstrated that mortality was associated with severe frailty (HR 2.63, 95%CI 2.45–2.80), moderate frailty (HR 2.05, 95%CI 1.96–2.14), and mild frailty (HR 1.45, 95%CI 1.39–1.51), independent of age, gender, and population sector. Among patients aged 90 years and over, no significant differences in cumulative survival were found between those with moderate and severe frailty (*p* = 0.408).

**Conclusions:**

Frailty is prevalent among community-dwelling Israeli older adults aged 75 years and over, and it is associated with long-term mortality. Considering its association with long-term mortality across frailty levels until the age of 90, early identification and intervention for frailty are recommended within this population. Policymakers should consider the use of the cumulative deficit method for evaluating frailty at both the population health and clinical levels.

## Introduction

Population aging poses a challenge to healthcare systems worldwide. Significant advancements in medicine and technology have led to a substantial increase in life expectancy. The segment of older adults aged 75 years and over is currently the fastest-growing segment of the population. In 2020, there were 23 million Americans aged 75 and over; by 2050, this segment is expected to surpass 46 million Americans [1]. This demographic shift will also affect Israel, as 4.9% of its population, i.e., over 450,000 people, are currently aged 75 years and over [2]. This creates an urgent need to direct Israeli healthcare system resources to those who are most at risk of adverse clinical outcomes and death.

Age itself is not the bearer of risk; rather, age-associated diseases and health deficits create vulnerability also known as frailty. Frailty is a powerful indicator of disability, loss of independence, hospitalization, and death [3, 4]. Among community-dwelling older adults aged 70 years and over around the world, the prevalence of frailty ranges between 25% and 61% [5]. In Israel, the prevalence of frailty has been previously studied only in small-scale studies [6–9], with reported rates ranging from 4.9 to 46%. However, it has not been investigated in a large, nationwide, unselected population of community-dwelling older adults.

Due to variations in frailty prevalence resulting from diverse definitions and regional disparities across studies [5, 10, 11], conventional prediction models may lose accuracy when they rely merely on age [12]. However, models predicting mortality based on frailty as deficit accumulation, defined as a simple count of age-associated diseases and health deficits, may offer greater accuracy in large populations of older adults, as observed in American veterans [13]. We sought to demonstrate the potential utility of this approach within a large database of community-dwelling older adults aged 75 years and over in Israel by studying the prevalence of frailty and its association with long-term mortality in this population.

## Methods

This was a retrospective cohort study conducted at Meuhedet Health Maintenance Organization (HMO), the third largest integrated healthcare service provider and payer system in Israel, which serves more than 1,276,000 people, approximately 13.8% of the 9,250,000 Israelis [14]. The study was approved by the Meuhedet HMO Helsinki ethics committee.

### Study design and population

The study cohort included patients aged 75 years and over on January 1st, 2014, or those who turned 75 years between January 1st, 2014, and September 30th, 2019. For patients aged 75 and over on January 1st, 2014, the index date was set as that date; patients who turned 75 during the study period had their index date set as their 75th birthday. All patients remained continuously insured by Meuhedet HMO throughout the study period. Follow-up started at the index date and continued until September 30th, 2021, or death. To ensure a minimum follow-up period of two years, only patients who turned 75 by September 30th, 2019, were included in the cohort. The frailty index (FI) scores were calculated at the index date, utilizing clinical data extracted from a maximum preceding 10-year period, with each diagnosis required to appear at least once.

### Data collected

The Meuhedet HMO data are stored in a data warehouse that integrates hospital and community medical records, imaging, laboratory results, and pharmaceutical records. The extracted data included age, gender, population sectors, and chronic co-morbidities. Population sectors were defined based on the patient’s primary care clinic address. The three population sectors recorded in the Meuhedet HMO are non-Orthodox Jews, Orthodox Jews, and Arabs. The outcome was all-cause mortality which was obtained from the Israeli General Registry Office database.

### Frailty index definition

Frailty was defined according to the cumulative deficit method [4, 13, 15] using ICD-9 codes of chronic diseases and age-related health deficits extracted from medical charts. A previously published FI by Orkaby et al. [13] underwent revision and adaptation to the Meuhedet HMO database. The original index included 31 variables; however, after revising the code definitions only 28 variables were included in the FI calculation. Three variables were excluded: “use of durable medical equipment” due to absence of current procedural terminology codes in the data; “chronic pain” and “failure to thrive” as they lacked proper definitions in the data. The total number of chronic diseases and age-related health deficits for each patient was divided by the total number of 28 variables, yielding a FI score between 0 and 1 [16]. For example, a patient with 10 out of 28 possible chronic diseases and age-related health deficits had a FI of 10/28 = 0.36. Following Orkaby et al. [13], frail patients were those with FI ≥ 0.21, and non-frail patients had FI ≤ 0.2. Frailty was further categorized into three severity levels: mild (FI 0.21–0.30), moderate (FI 0.31–0.40), and severe (FI > 0.4). Chronic diseases included chronic kidney disease, all types of arthritis, atrial fibrillation or flutter, active cancer excluding skin carcinomas, cerebrovascular disease, ischemic heart disease, type 2 diabetes mellitus, chronic gastrointestinal diseases, heart failure, essential hypertension, obstructive lung diseases, osteoporosis, Parkinson’s disease, peripheral vascular disease, chronic thyroid diseases, anxiety, depression and other mood disorders, anemia, dementia, recurrent falls, peripheral neuropathy, and fatigue. Age-related health deficits were related to functional status (e.g., gait abnormalities, muscular wasting), sensory impairment (e.g., hearing impairment, visual impairment), geriatric syndromes (e.g., urinary incontinence, fecal incontinence), and weight loss in the previous year. All variables included in the Frailty Index were dichotomous, determined by the presence or absence of a diagnosis in a patient’s history, thus eliminating the presence of missing values in our analysis.

### Statistical analysis

Categorical variables were reported as numbers and percentages and were compared between groups by using the Chi-square test and Fisher’s exact test. Continuous variables with a non-normal distribution were reported as median and interquartile range (IQR). Kaplan-Meier curves were employed to compare cumulative survival among patients with varying frailty severities. The Benjamini-Hochberg procedure was applied to present multiple comparison tables. A univariate Cox proportional hazards regression model was used to assess the association of mortality with the variables frailty, age, gender, and population sector. A multivariate Cox proportional hazards regression model was used to identify independent associations with mortality for these same variables. A two-tailed *p* < 0.05 was considered statistically significant. All statistical analysis was performed using R Statistical Software (version 4.1.0; R Foundation for Statistical Computing, Vienna, Austria).

## Results

The cohort included 43,737 older adults aged 75 years and over at the index date, including 24,437 (55.9%) females and 19,300 (44.1%) males. The median age was 77 years (IQR 75–82 years). The majority of patients were non-Orthodox Jews (*n* = 35,515, 81.2%). The three most prevalent chronic diseases and age-related health deficits were essential hypertension, arthritis, and hearing impairment (Table 1). The median number of chronic diseases and age-related health deficits was 5 (IQR 3–7).

**Table 1:** Demographic and clinical characteristics of the entire cohort

**Demographics**	
Age at index date, years, median [IQR]	77 [75, 82]
Male gender, n (%)	19,300 (44.1)
**Sectors**	
Non-Orthodox Jews, n (%)	35,515 (81.2)
Orthodox Jews, n (%)	5,780 (13.2)
Arabs, n (%)	2,442 (5.6)
**Frailty**	
None, n (%)	24,341 (55.7)
Mild, n (%)	12,260 (28.0)
Moderate, n (%)	5,533 (12.6)
Severe, n (%)	1,603 (3.7)
**Chronic diseases and age-related health deficits**	
Essential hypertension, n (%)	36,205 (82.8)
Arthritis, n (%)	25,677 (58.7)
Hearing impairment, n (%)	17,514 (40.0)
Coronary artery disease, n (%)	13,385 (30.6)
Type 2 diabetes mellitus, n (%)	13,230 (30.2)
Obstructive lung disease, n (%)	12,132 (27.7)
Peripheral vascular disease, n (%)	12,093 (27.6)
Anxiety, n (%)	11,640 (26.6)
Depression and mood disorders, n (%)	10,980 (25.1)
Chronic thyroid disease, n (%)	9,408 (21.5)
Active cancer, n (%)	9,097 (20.8)
Cerebrovascular disease, n (%)	9,110 (20.8)
Gastrointestinal disease, n (%)	8,388 (19.2)
Chronic kidney disease, n (%)	6,793 (15.5)
Atrial fibrillation or flutter, n (%)	6,389 (14.6)
Dementia, n (%)	5,560 (12.7)
Heart failure, n (%)	5,280 (12.1)
Anemia, n (%)	4,887 (11.2)
Parkinson’s disease, n (%)	3,600 (8.2)
Muscular wasting, n (%)	3,462 (7.9)
Gait abnormality, n (%)	3,416 (7.8)
Vision impairment, n (%)	3,151 (7.2)
Osteoporosis, n (%)	2,670 (6.1)
Fecal or urinary incontinence, n (%)	2,428 (5.6)
Peripheral neuropathy, n (%)	1,983 (4.5)
Recurrent falls, n (%)	1,653 (3.8)
Fatigue, n (%)	1,362 (3.1)
Weight loss in the previous year, n (%)	1,240 (2.8)

At the index date, 24,341 (55.7%) older adults were not frail, while 19,396 (44.3%) older adults were frail: 12,260 (28.0%) mildly frail, 5,533 (12.7%) moderately frail and 1,603 (3.7%) severely frail (Table 1).

The prevalence of frailty was higher in females (*n* = 11,353, 46.5%) than in males (*n* = 8,043, 41.7%) (*p* < 0.001). Among females, rates of mild, moderate, and severe frailty were 29.5% (*n* = 7,219), 13.2% (*n* = 3,215), and 3.8% (*n* = 919), respectively, while for males, these rates were 26.1% (*n* = 5,041), 12% (*n* = 2,318), and 3.5% (*n* = 684). Females had a median age of 77 (IQR 75-82.9 years), slightly higher than males at 76.3 (IQR 75-81.4 years) (*p* < 0.001). The prevalence of frailty was higher in non-Orthodox Jews (*n* = 16,147, 45.5%) than in Orthodox Jews (*n* = 2,280, 39.4%) and Arabs (*n* = 969, 39.7%) (*p* < 0.001). Non-Orthodox Jews and Orthodox Jews were older (median age 77 years, IQR 75–82 years) than Arabs (median age 75, IQR 75–79 years) (*p* < 0.001). The median FI was 0.18 (IQR 0.11–0.25) in all population sectors.

The prevalence of all chronic diseases and age-related health deficits increased significantly with frailty severity (*p* < 0.001). The most notable increases were observed in depression and other mood disorders, as well as in coronary artery disease, peripheral vascular disease, and heart failure (Table 2).

**Table 2:** Prevalence of chronic diseases and age-related health deficits across frailty levels

	Non- frail (*n* = 24,341)	Mild Frailty (*n* = 12,260)	Moderate Frailty (*n* = 5,533)	Severe Frailty (*n* = 1,603)
Frailty score, Median [IQR]	0.12 [0.07–0.16]	0.25 [0.21–0.27]	0.34 [0.32–0.36]	0.45 [0.43–0.48]
Essential hypertension, n (%)	17,925 (73.6)	11,343 (92.5)	5,349 (96.7)	1,588 (99.0)
Arthritis, n (%)	11,072 (45.5)	8,764 (71.5)	4,447 (80.4)	1,394 (87.0)
Hearing impairment, n (%)	6,980 (28.7)	6,144 (50.1)	3,251 (58.8)	1,139 (71.0)
Coronary artery disease, n (%)	4,016 (16.5)	4,836 (39.4)	3,325 (60.1)	1,208 (75.3)
Type 2 diabetes mellitus, n (%)	4,593 (18.9)	4,682 (38.2)	2,875 (52.0)	1,080 (67.4)
Obstructive lung disease, n (%)	4,155 (17.1)	4,312 (35.2)	2,688 (48.6)	977 (61.0)
Peripheral vascular disease, n (%)	3,490 (14.3)	4,436 (36.2)	3,045 (55.0)	1,122 (70.0)
Anxiety, n (%)	3,527 (14.5)	4,504 (36.7)	2,613 (47.2)	996 (62.1)
Depression, mood disorders, n (%)	2,722 (11.2)	4,247 (34.6)	2,881 (52.1)	1,130 (70.5)
Chronic thyroid disease, n (%)	3,355 (13.8)	3,366 (27.5)	1,949 (35.2)	738 (46.0)
Active cancer, n (%)	3,550 (14.6)	3,219 (26.3)	1,718 (31.0)	610 (38.1)
Cerebrovascular disease, n (%)	2,307 (9.5)	3,422 (27.9)	2,417 (43.7)	964 (60.1)
Gastrointestinal disease, n (%)	2,616 (10.7)	3,185 (26.0)	1,891 (34.2)	696 (43.4)
Chronic kidney disease, n (%)	2,091 (8.6)	1,756 (14.3)	2,021 (36.5)	925 (57.7)
Atrial fibrillation or flutter, n (%)	1,563 (6.4)	2,303 (18.8)	1,747 (31.6)	776 (48.4)
Dementia, n (%)	1,307 (5.4)	1,916 (15.6)	1,627 (29.4)	710 (44.3)
Heart failure, n (%)	839 (3.4)	1,796 (14.6)	1,783 (32.2)	862 (53.8)
Anemia, n (%)	1,166 (4.8)	1,719 (14.0)	1,369 (24.7)	633 (39.5)
Parkinson’s disease, n (%)	849 (3.5)	1,293 (10.5)	989 (17.9)	469 (29.3)
Muscular wasting, n (%)	729 (3.0)	1,115 (9.1)	1,071 (19.3)	547 (34.1)
Gait abnormality, n (%)	564 (2.3)	1,142 (9.3)	1,124 (20.3)	586 (36.6)
Vision impairment, n (%)	1,081 (4.4)	1,129 (9.2)	673 (12.2)	268 (16.7)
Osteoporosis, n (%)	821 (3.4)	1,017 (8.3)	602 (10.9)	230 (14.4)
Incontinence (fecal\urinary), n (%)	473 (1.9)	817 (6.7)	769 (13.9)	369 (23.0)
Peripheral neuropathy, n (%)	240 (1.0)	620 (5.1)	728 (13.2)	395 (24.7)
Recurrent falls, n (%)	442 (1.8)	624 (5.1)	418 (7.5)	169 (10.6)
Fatigue, n (%)	369 (1.5)	513 (4.2)	345 (6.2)	135 (8.4)
Weight loss, previous year, n (%)	288 (1.2)	418 (3.4)	354 (6.4)	180 (11.2)

The median follow-up time was 5.9 years (IQR 3.4–7.8 years). Overall, 15,064 (34.4%) older adults died during the follow-up period. The median age of death was 86 years (IQR 81–91 years). Overall, 6,186 (25.4%) non-frail older adults and 8,878 (45.8%) frail older adults died during the follow-up period: 4,782 (39.0%) among the mildly frail, 3,016 (54.5%) among the moderately frail and 1,080 (67.4%) among the severely frail.

**Fig. 1:**
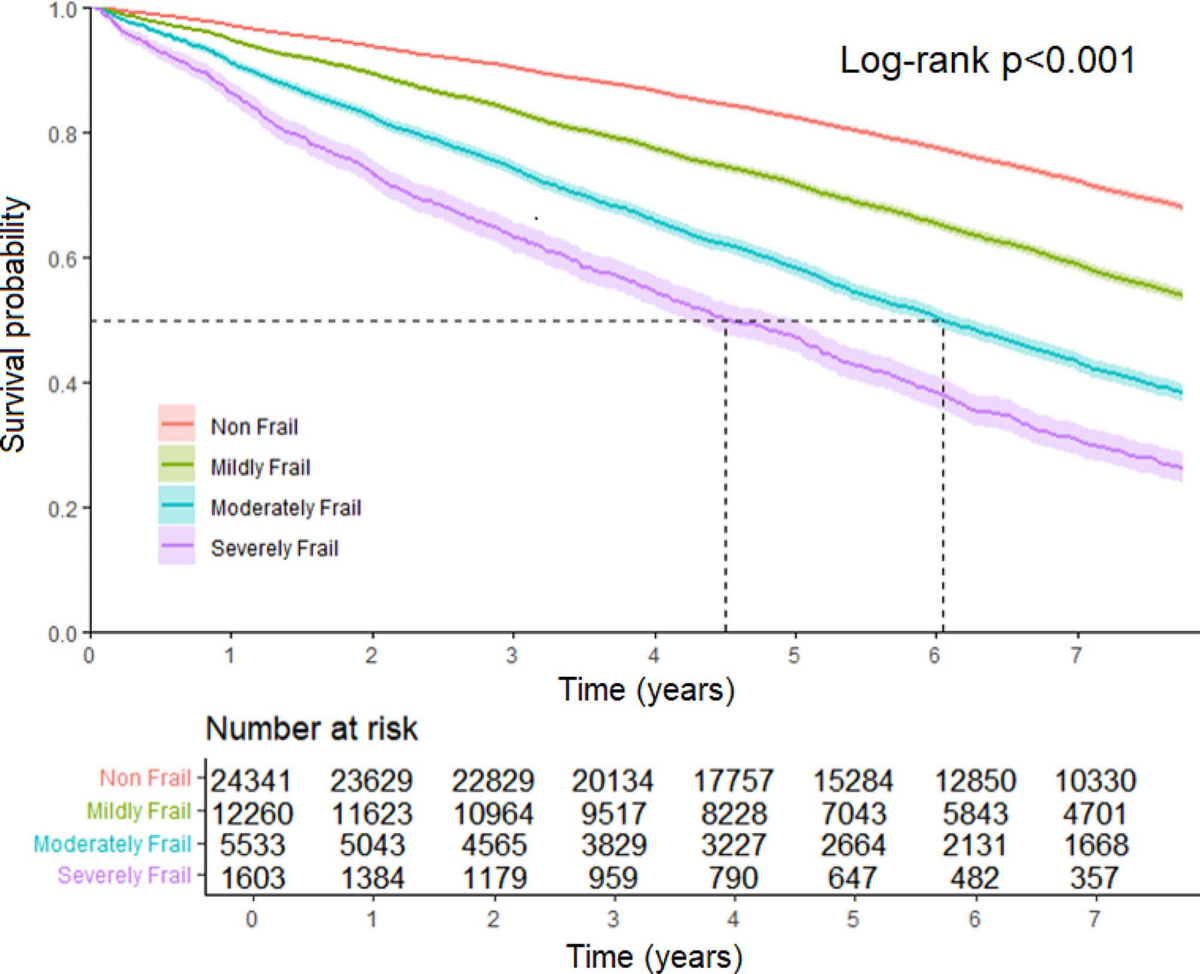
Kaplan-Meier survival curves across frailty levels

**Fig. 2:**
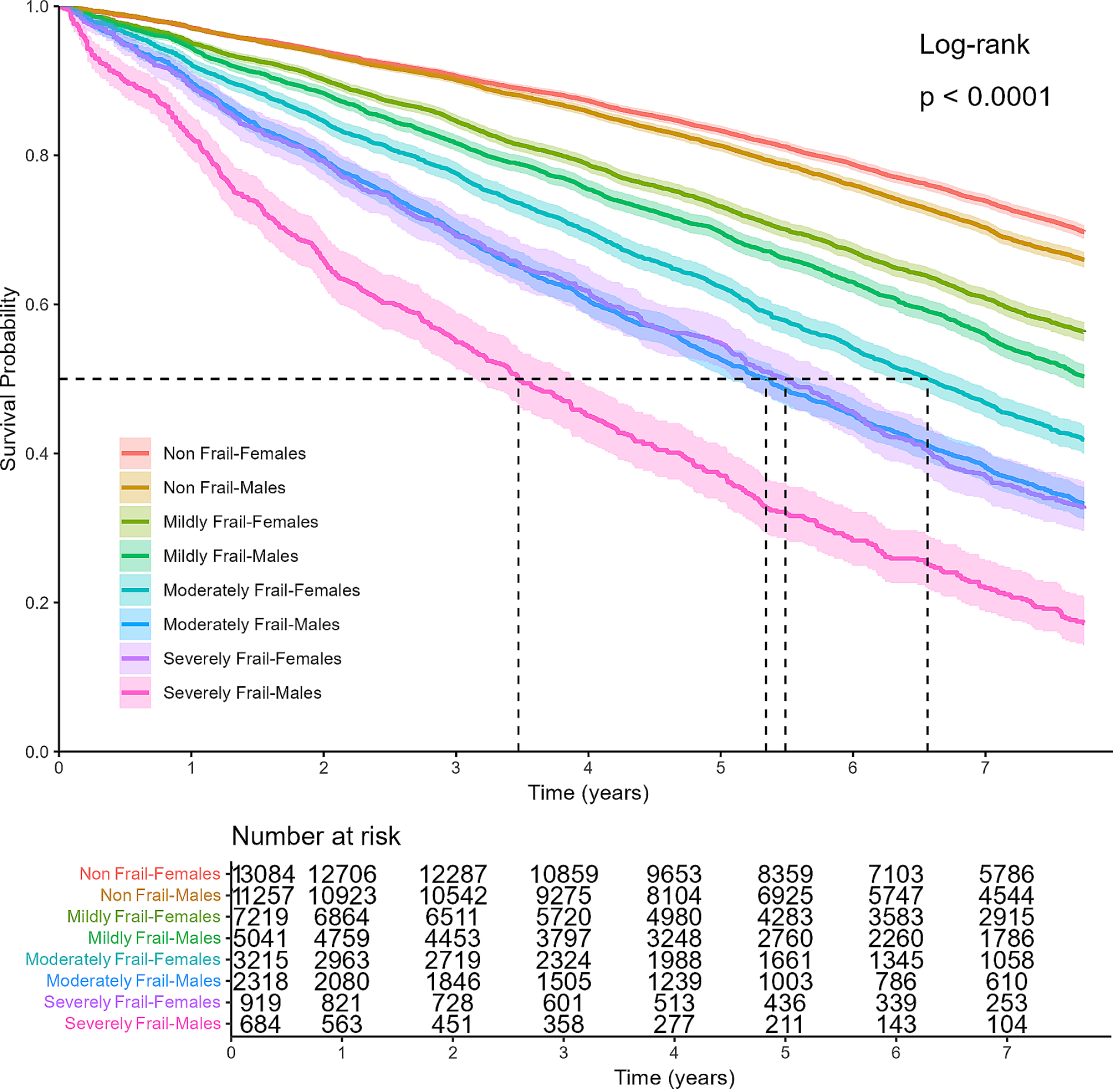
Kaplan-Meier survival curves across frailty levels, stratified by gender

Kaplan-Meier survival curves demonstrated that cumulative survival decreased significantly by frailty severity. The median survival of moderately frail older adults was 6.04 years (95%CI 5.89–6.24 years), and the median survival of severely frail older adults was 4.50 years (95%CI 4.23–4.98 years) (Fig. 1). Figure 2 illustrates Kaplan-Meier curves showing mortality rates stratified by gender across different frailty levels. Females exhibit lower mortality rates compared to males across all frailty levels. Notably, the mortality rates for moderately frail males and severely frail females appear comparable (*p* = 0.86).

**Fig. 3:**
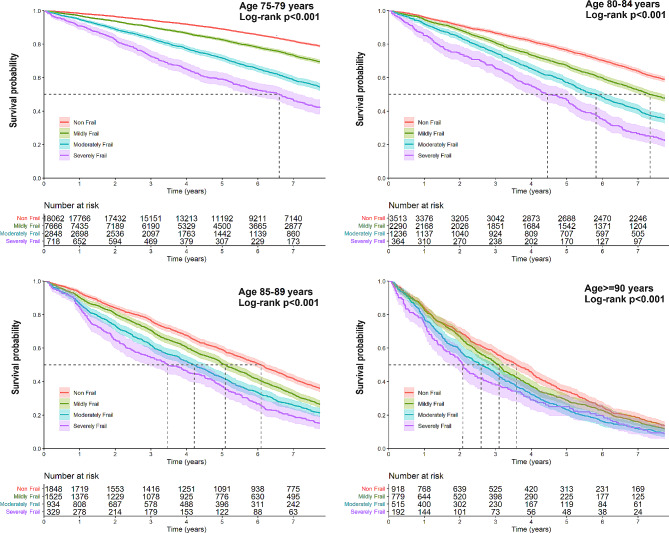
Kaplan-Meier survival curves across frailty levels, stratified by age groups

Kaplan-Meier survival curves also demonstrated a significant decrease in cumulative survival by frailty severity across all age groups. However, the distinctions in cumulative survival between mild, moderate, and severe frailty diminished with age, and these differences remained significant (*p* < 0.001) until the age of 90 years. For patients aged 90 years and over, no significant differences in cumulative survival were observed between those with moderate frailty and severe frailty (*p* = 0.408); the differences between patients with mild frailty and moderate/severe frailty were modest (*p* = 0.017 for both). The median survival of older adults aged 90 years and over was 3.10 years (95%CI 2.88–3.23 years) in mild frailty, 2.59 years (95%CI 2.30–2.97 years) in moderate frailty, and 2.08 years (95%CI 1.73–2.49) in severe frailty (Fig. 3).

**Fig. 4:**
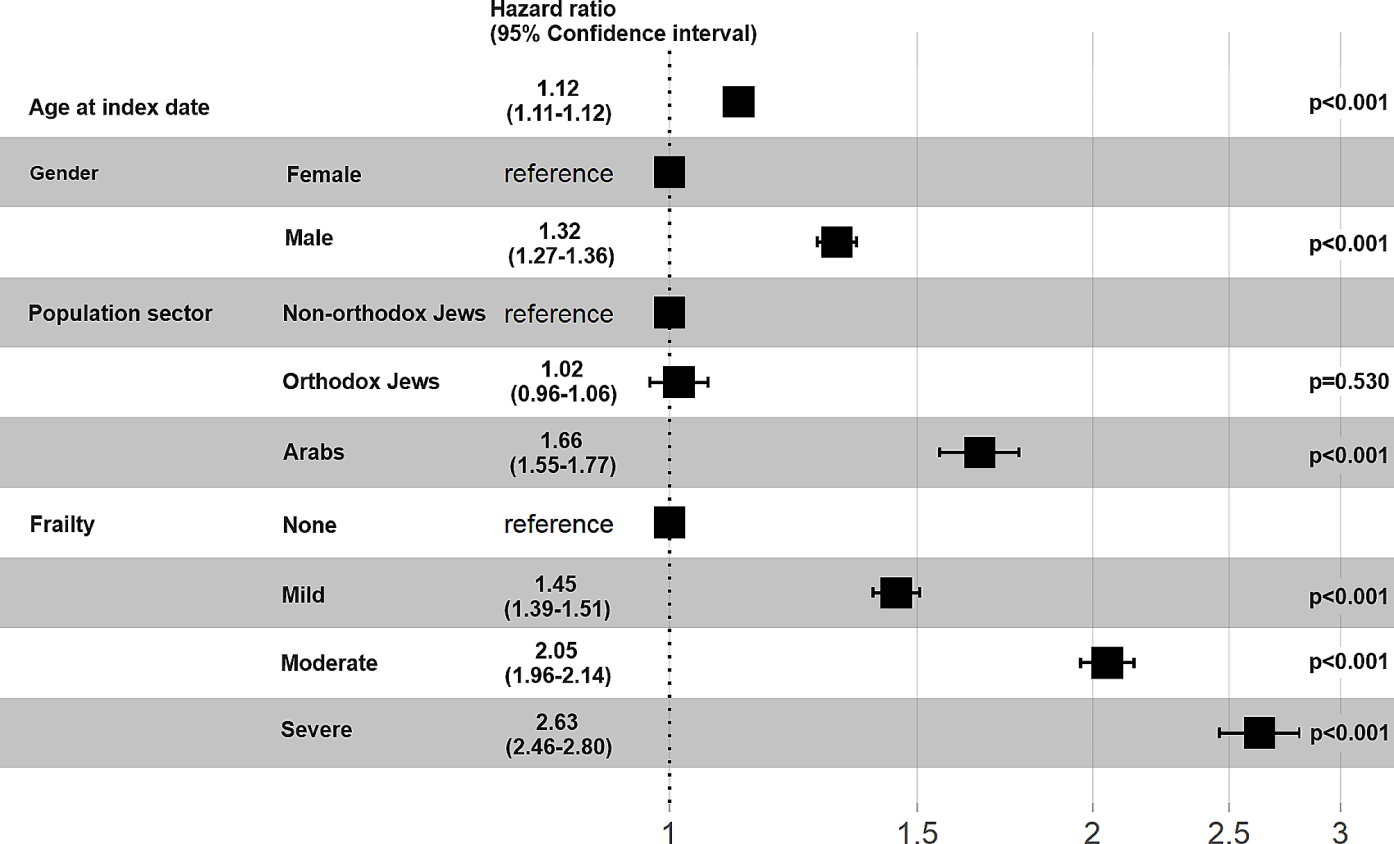
Independent associations with mortality in a multivariate cox proportional hazards regression model

A univariate Cox proportional hazards regression model demonstrated that, in comparison to survivors, deceased patients were characterized by older age, a higher proportion of males, Arabs, and all degrees of frailty severity (Table 3). In a multivariate Cox proportional hazards regression model, mortality was associated with severe frailty (HR 2.63, 95%CI 2.46–2.80), moderate frailty (HR 2.05, 95%CI 1.96–2.14), and mild frailty (HR 1.45, 95%CI 1.39–1.51), as well as with age at the index date (HR 1.12, 95%CI 1.11–1.12), male gender (HR 1.32, 95%CI 1.27–1.36), and the Arab sector (HR 1.66, 95%CI 1.55–1.77) (Fig. 4).

**Table 3:** Demographic and clinical characteristics by survival status - univariate cox analysis

	Survivors (*n* = 28,673)	Deceased (*n* = 15,064)	Hazard ratio (95% Confidence interval)	*p* value
**Demographics**				
Age at index date, years, median [IQR]	75 [75, 78]	82 [77, 87]	1.13 (1.12–1.13)	< 0.001
Male gender, n (%)	12,300 (42.9)	7,000 (46.5)	1.16 (1.12–1.19)	< 0.001
**Population sector**				
Non-Orthodox Jews, n (%)	23,414 (81.7)	12,101 (80.3)	reference	
Orthodox Jews, n (%)	3,796 (13.2)	1,984 (13.2)	1.02 (0.98–1.07)	0.344
Arabs, n (%)	1,463 (5.1)	979 (6.5)	1.36 (1.28–1.46)	< 0.001
**Frailty**				
None, n (%)	18,155 (63.3)	6,186 (41.1)	reference	
Mild, n (%)	7,478 (26.1)	4,782 (31.7)	1.64 (1.58–1.71)	< 0.001
Moderate, n (%)	2,517 (8.8)	3,016 (20.0)	2.62 (2.51–2.73)	< 0.001
Severe, n (%)	523 (1.8)	1,080 (7.2)	3.77 (3.53–4.02)	< 0.001

## Discussion

The main finding of the current analysis is the high prevalence of frailty among Israeli older adults aged 75 years and over: nearly half (44.3%) of this population was frail. Additionally, the prevalence of all chronic diseases and age-related health deficits increased significantly with advancing frailty – particularly in the case of depression and other mood disorders. Another significant finding of this analysis is the high mortality rates among frail Israeli older adults aged 75 years and over: nearly half (45.8%) of the patients died during the follow-up period, and mortality was independently associated with frailty severity. Furthermore, the differences in cumulative survival between mild, moderate, and severe frailty diminished with age. In patients aged 90 years and over, there were either modest differences or no differences in cumulative survival by frailty severity.

The prevalence of frailty in the general population of Israeli older adults and its association with mortality were previously studied in a small cohort of 840 community-dwelling older adults aged 85 years and over [6]. In that study, the prevalence of frailty was comparatively low (19.5%) in contrast to the current analysis, possibly because Jacobs et al. defined frailty using the phenotype method [3]. A meta-analysis conducted by Shamliyan et al. [7] reported that the mean prevalence of frailty in older adults aged 65 years and over, defined by the phenotype method, is 14%, whereas the mean prevalence of frailty defined by the cumulative deficit method is 24%. Buch et al. [8] also studied the prevalence of frailty in the general population of Israeli community-dwelling older adults; however, compared with the current analysis, their cohort was smaller (*n* = 1619), younger (aged 65 years and over), and did not include patients with moderate to severe dementia. Accordingly, the prevalence of frailty in their cohort was much lower (4.9%), and its association with mortality was not reported. Goshen et al. [9], using a frailty index based on the cumulative deficit method, reported a frailty prevalence of 46% in a small cohort (*n* = 479), which is comparable to our findings, despite their cohort being younger (aged 65 years and over). Indeed, the prevalence of frailty in the current analysis is comparable to worldwide studies which use the cumulative deficit method – ranging between 25% and 61% in older adults aged 70 years and over [5].

According to the Israeli central bureau of statistics, the mean life-expectancy of Israeli women and men aged 75 years and over during the study period ranged from 10 to 13.7 years and 8.9 to 12.1 years, respectively [17]. Survival rates in the current analysis were lower. Almost half (45.8%) of the frail adults aged 75 years and over died during the follow-up period. The median survival of moderately and severely frail older adults was 6.04 years and 4.50 years, respectively. These findings carry clinical and financial implications. Preventive therapies with a 5-year time frame to show benefit, such as statin therapy for primary cardiovascular prevention, may be irrelevant for Israeli older adults aged 75 years and over with moderate to severe frailty [8]. Studies indicate that between a third and half of the oldest-old in Israel take statins [18, 19]. Many of these patients are likely frail and may not benefit from this therapy and even be harmed by it [20]. Implementing an automated system that presents the FI of older patients to primary physicians may help prevent unnecessary and potentially harmful treatments.

The differences in cumulative survival between mild, moderate, and severe frailty decrease with age in the current analysis, in concordance with findings from previous studies [9, 21]. However, the association between frailty severity and mortality has been seldom studied in patients aged 90 years and over. According to Gu et al. [22], 3-year mortality rates are almost similar in Chinese men aged 90–99 years and 100–109 years across frailty levels. Additionally, according to Romero-Ortuno et al. [23], there are no differences in cumulative survival between European men aged 90 years and over with moderate frailty and severe frailty. These findings are consistent with the current analysis. While frailty emerges as a stronger predictor of mortality than chronological age, as observed in the current analysis and others [22, 23], it is possible that in very old patients, the small physiological reserve plays a more significant role than comorbidity. Gender-stratified analysis revealed that females exhibit higher rates of frailty across all levels. However, paradoxically, females demonstrate lower mortality rates across all levels of frailty, as previously described [24].

Depression and frailty are prevalent conditions in older age, yet the interplay between them remains unclear. It is uncertain whether depression contributes to the emergence of frailty, vice versa, or if these two conditions coexist independently. Collard et al. [25] suggest that frailty predicts depression and is associated with lower rates of depression remission. According to Vaughan et al. [26], the prospective relationship between depressive symptomatology and increased risk of frailty is robust; however, the opposite relationship is less definitive. Almeida et al. [27] indicate that depression is associated with increased mortality in older adult men, and this excess mortality is strongly associated with frailty. The current findings support the theory that depression facilitates the appearance of frailty, given that the prevalence of depression significantly increases with frailty severity more than other chronic diseases.

The current study carries important implications for health policy across various domains. At the population health level, an automated frailty index derived from the cumulative deficit model can contribute to planning, prioritizing, and allocating resources to meet the needs of this high-risk patient group. At the clinical level, incorporating a frailty index into the workflow of primary care physicians might enhance informed decision-making in various domains, including preventive medicine, major surgery, and cancer therapies. However, when considering such an option, it is important to acknowledge the differing approaches clinicians have toward the use of frailty indexes, as demonstrated in a recent study by Seeley et al. [28]. While the majority of practitioners expressed overall support for formalized frailty assessment, others had concerns regarding the accuracy and interpretation of such scores at the patient level. Presenting frailty data in the medical chart could potentially stigmatize older adults and even deprive them of useful therapy. Furthermore, the absence of consideration for quality of life or other outcomes highlights potential limitations in relying solely on the frailty index for clinical decision-making.

The main strength of this study lies in its substantial size, conducted using a national computerized database that encompassed all medical records from a diverse and unselected patient population. The main limitation of the study arises from relying solely on formal diagnoses, documented for clinical purposes rather than being specifically tailored for research. This approach may introduce information biases, particularly considering the potential misclassification associated with formal diagnoses and the exclusion of unknown diagnoses. Another limitation is the potential for residual confounding by variables not adjusted for, such as social support, smoking status, alcohol consumption, utilization of health services, and adherence to screening tests. These unmeasured factors could introduce bias into our findings and impact the interpretation of the results. Another limitation is the FI being calculated only at the index date. Accordingly, conclusions regarding the rate of frailty changes during the follow-up period and their impact on mortality could not be drawn and should be addressed in future analyses. Finally, the external validity of our study may be influenced by its overrepresentation of Orthodox Jews, comprising 13.2% of the cohort, which may limit its generalizability to the broader Israeli population with different demographic compositions.”

## Conclusion

In conclusion, frailty is prevalent among the general population of community-dwelling Israeli older adults aged 75 years and over, and it is associated with long-term mortality across frailty levels until the age of 90. To ensure early diagnosis an intervention in this population, policymakers should consider incorporating automatic frailty index score calculations into electronic medical records using the cumulative deficit method. Consequently, both general practitioners and geriatricians will be prompted to address frailty, recognizing its potential association with mortality in older adults. Additionally, the identification and management of depression and other mood disorders should be prioritized in this population.

## Data Availability

The data that support the findings of this study originate from a large Israeli HMO. Restrictions apply to the availability of these data and they are therefore not publicly available.

## Abbreviations

CI: Confidence interval
FI: Frailty index
HMO: Health maintenance organization
HR: Hazard ratio
IQR: Interquartile range
